# Randomized controlled trial on cardiovascular risk management by practice nurses supported by self-monitoring in primary care

**DOI:** 10.1186/1471-2296-13-90

**Published:** 2012-09-04

**Authors:** Ans H Tiessen, Andries J Smit, Jan Broer, Klaas H Groenier, Klaas van der Meer

**Affiliations:** 1 Department General Practice, University Medical Centre, Groningen, the Netherlands; 2 Department Internal Medicine, University Medical Centre, Groningen, the Netherlands; 3 Municipal Public Health Service Groningen, Groningen, the Netherlands

**Keywords:** Primary health care, Arteriosclerosis, Cardiovascular diseases, Prevention and control, Self-management, Risk factors

## Abstract

**Background:**

Treatment goals for cardiovascular risk management are generally not achieved. Specialized practice nurses are increasingly facilitating the work of general practitioners and self-monitoring devices have been developed as counseling aid. The aim of this study was to compare standard treatment supported by self-monitoring with standard treatment without self-monitoring, both conducted by practice nurses, on cardiovascular risk and separate risk factors.

**Methods:**

Men aged 50–75 years and women aged 55–75 years without a history of cardiovascular disease or diabetes, but with a SCORE 10-year risk of cardiovascular mortality ≥5% and at least one treatable risk factor (smoking, hypertension, lack of physical activity or overweight), were randomized into two groups. The control group received standard treatment according to guidelines, the intervention group additionally received pro-active counseling and self-monitoring (pedometer, weighing scale and/ or blood pressure device). After one year treatment effect on 179 participants was analyzed.

**Results:**

SCORE risk assessment decreased 1.6% (95% CI 1.0–2.2) for the control group and 1.8% (1.2–2.4) for the intervention group, difference between groups was .2% (−.6–1.1). Most risk factors tended to improve in both groups. The number of visits was higher and visits took more time in the intervention group (4.9 (SD2.2) vs. 2.6 (SD1.5) visits p < .001 and 27 (P_25_ –P_75_:20–33) vs. 23 (P_25_ –P_75_:19–30) minutes/visit p = .048).

**Conclusions:**

In both groups cardiovascular risk decreased significantly after one year of treatment by practice nurses. No additional effect of basing the pro-active counseling on self-monitoring was found, despite the extra time investment.

**Trial registration:**

trialregister.nl NTR2188

## Background

Cardiovascular diseases are the most important cause of death worldwide [1]. Preventive treatment of these diseases is targeted at individuals with an elevated cardiovascular risk, based on a combined risk factor approach [2,3]. However, treatment goals for cardiovascular risk factors are generally not achieved [4], which underlines the importance of improving the efficacy of treatment and follow-up programs.

One of the approaches in the Anglo-Dutch health care system has been to train specialized practice nurses to assist the general practitioner (GP). They integrate lifestyle counseling and drug treatment according to the guidelines and achieve promising results, as was shown in several international studies on cardiovascular risk management [5-7].

Another development is the use of self-monitoring (e.g. blood pressure devices, pedometers and standardized weighing scales for use at home) in lifestyle and pharmaceutical counseling. The use of each of these devices has demonstrated improvements on the specific risk factors [8-11] and in the case of home blood pressure measurements even led to a decreased use of medication [12]. Self-monitoring of *combined* parameters is already successfully being used in chronic heart failure patients [13].

The common approach taken in the Anglo-Dutch-Scandinavian health care system consists of integrated lifestyle counseling and drug treatment for individuals with an elevated cardiovascular risk [2,3]. The height of the risk is estimated based on a combination of different risk factors (hypertension, hypercholesterolemia, overweight, smoking and lack of physical activity) and treatment is aimed at the individual combination of these risk factors. To our knowledge, no study has yet investigated the combined intervention of this cardiovascular risk management approach, supported by self-monitoring equipment, conducted by trained practice nurses in general practices, even though such an approach is in line with the identified developments and might be effective.

The research question that the SPRING study (Self-monitoring and Prevention of RIsk factors by Nurse practitioners in the region of Groningen) aimed to answer, was: is cardiovascular risk management according to the Dutch GP’s Guideline [3], supported by self-monitoring, more effective than standard cardiovascular risk management according to the same guideline, in primary care? Effectiveness of both treatment strategies was evaluated after one year on SCORE (Systematic Coronary Risk Evaluation) 10-year risk of fatal cardiovascular disease [14] and on separate risk factors.

## Methods

### Eligibility criteria for participants

Eligible participants were men aged 50–75 years and women aged 55–75 years. As women on average start to have an elevated cardiovascular risk at an older age then men, age inclusion was adapted to avoid major sex imbalance in other risk factors. Inclusion furthermore required a SCORE cardiovascular risk assessment ≥5% and an indication for treatment for at least one risk factor (Table 1). Individuals with hypercholesterolemia *alone* were not included, because treatment for this was equal for both study groups (Table 2). Exclusion criteria were: a history of cardiovascular disease, diabetes mellitus, thyroid dysfunction or a seriously diminished life expectancy (estimated <2 years). Written informed consent was given by all participants. The SPRING study was approved by the Medical Ethics Review Committee of the University Medical Centre Groningen (reference number 2007/232).

**Table 1:** Risk factor cut off points according to the Dutch GP guideline

**Risk factor**	**Cut off point for recommendation as a treatment goal**
**Overweight**	BMI ≥25 kg/m^2^ or waist circumference >80 cm♀/>94 cm♂
**Smoking**	≥1 item/day
**Physical inactivity**	<30 minutes moderately intensive physical activity on ≥5 days/week
**Hypertension**	systolic blood pressure ≥140 mmHg and ≥1 additional risk factor*
**Hypercholesterolemia**	LDL >2,5 mmol/l and ≥1 additional risk factor*

* additional risk factors were a SCORE risk assessment ≥ 10%, positive family history for cardiovascular disease (<60 years in first degree relative), serum creatinine. >115-150♂/107-150♀ umol/l, waist circumference >88 cm♀/ >102 cm♂ and BMI > 30 kg/ m^2^.

**Table 2:** Treatment programs and follow-up schedules for the different risk factors in both treatment groups

**Risk factor**	**Control group**	**Intervention group**
**Overweight**	· One single advice + standard information leaflet from Dutch GP society.	· Intensive counseling and feedback on energy intake and expenditure, supported by food diary, home weight scale (Microlife WS 80), step diary and pedometer (Yamax digiwalker SW-200).
	· More counseling or referral only on patient’s request	· Follow-up 3 times at monthly intervals and after that at 3-monthly intervals.
**Smoking**	· One single advice + standard information leaflet from Dutch GP society.	· Intensive counseling and feedback based on Stage of Change, Minimal Intervention Strategy and Dutch GP guideline.
	· More counseling or referral only on patient’s request	· Follow-up monthly until planned date of quitting and after that at increasing intervals.
Physical inactivity*	· One single advice + standard information leaflet from Dutch GP society.	· Intensive counseling and feedback on increasing physical activity, supported by step diary and pedometer (Yamax digiwalker SW-200).
	· More counseling or referral only on patient’s request	· Follow-up 3 times at monthly intervals en after that at 3-monthly intervals.
**Hypertension**	Medication and follow-up according to Dutch GP guideline. Follow-up: monthly (hypert.) /3-monthly (hyperchol.) until optimal treatment.	Same as control group except feedback based on home measurements (Microlife Watch BP Home).
**Hypercholesterolemia**		Same as control group.

* If participants also had an indication to lose weight, increase of physical activity was not a separate goal.

### Recruitment

**Figure 1:**
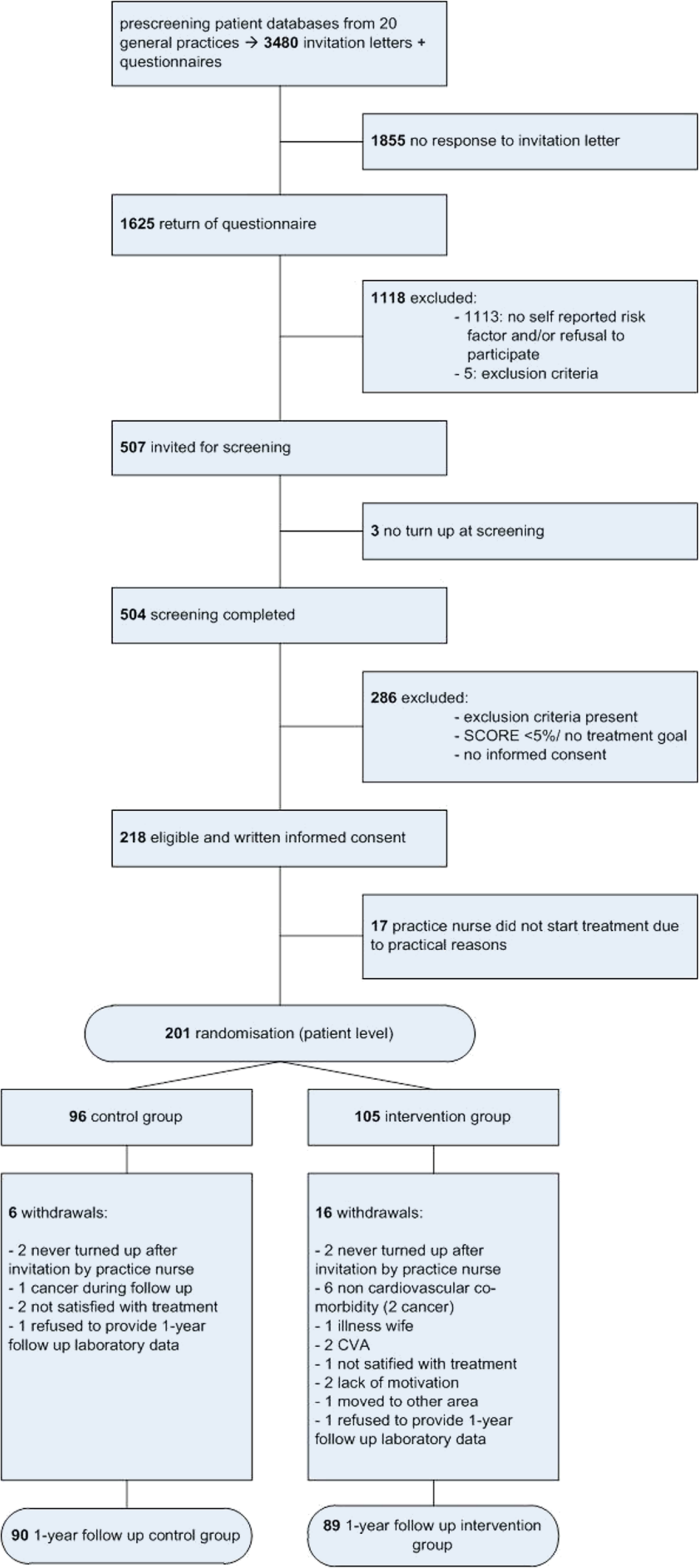
Flow of patients through the SPRING study.

20 general practices at 15 locations in the Netherlands participated. From each practice patient database approximately 200 individuals meeting age requirements and without registered exclusion criteria were randomly selected. The GP excluded individuals with a diminished life expectancy. Between April and December 2008, 3480 selected individuals received an invitation letter with a questionnaire (Figure 1). This questionnaire contained items on smoking, BMI >24 kg/m^2^ and physical activity <150 minutes/week (2 questions based on the Short Questionnaire to Assess Health-enhancing Physical Activity, SQUASH) [15]. Individuals were invited for the screening if they were interested in participating and had at least one risk factor. Between June 2008 and August 2009 a trained research team collected the following screening data at the different practice locations. 

*Physical examination:* Body weight, height and waist circumference were collected. Blood pressure was measured two times on each arm and this was repeated on a different day on the arm with the highest values. The mean value from this arm was used.

*Medical history:* Patient reported cardiovascular risk factors, medication use, medical history and family history were recorded.

*Blood test:* After an overnight fast serum glucose, lipids, thyroid, liver and kidney function were analyzed. If this suggested diabetes, familial hypercholesterolemia or thyroid dysfunction, individuals were excluded and the GP was informed. With other abnormal results it was decided by mutual agreement with the GP whether participation was safe or not.

The SCORE risk assessment was calculated based on sex, age, smoking status, systolic blood pressure and total/HDL cholesterol, using a computer program (Dutch GP Society ”Consultwijzer”, version December 2007). 65 is the maximum age for this risk calculator and was used for all participants aged ≥ 65 years. Treatment goals were individually determined (Table 1), based on risk factors, irrespective of current medication. Baseline data consisted of screening data, completed with a questionnaire, containing items on general characteristics and the standardized RAND-36 health survey and SQUASH [15,16].

Participants were allocated 1:1 to two parallel unpaired treatment groups, using computer generated random numbers. Randomization was at patient level.

### Intervention

General practices could participate if a practice nurse was working in the practice, which is the most current situation in Dutch general practices (2006: 70% of the practices) [17]. Practices were located in the northern part of the Netherlands, both at the city of Groningen and in smaller cities/villages. Fourteen practice nurses participated; ten of them were educated as nurses and four were originally GP-assistants with additional education as practice nurse. The practice nurses followed a specially developed training program, consisting of five sessions lasting four hours each. The training involved cardiovascular risk calculation, treatment guidelines and adapted motivational interviewing. Motivational interviewing is “a collaborative, person-centered form of guiding to elicit and strengthen motivation for change” [18]. This method, in which a client’s readiness to change, ambivalence and motivation are assessed and goal setting and changing of unhealthy behavior are reinforced, in collaboration with the client, has proven to be effective in various settings [18-21]. Closely related to this is the stage-of-change concept, which is used in the minimal intervention strategy for smoking cessation [22,23].

All patients received counseling regarding cardiovascular risk from practice nurses trained in motivational interviewing techniques and in the intervention group this counseling was based on self-monitoring results (pedometer, weighing scale and/ or blood pressure device). Table 2 shows treatment and follow-up programs for both study groups. Visits took place at the general practices. During the first visit, the individual SCORE risk assessment, present risk factor(s) and resulting treatment goals were discussed in both groups.

For the control group, follow-up visits were planned according to the Dutch GP guideline in case of hypertension and/or hypercholesterolemia [3].

For the intervention group, treatment for *all* present risk factors was pro-actively offered. The order in which the treatments for the different risk factors were started depended on preference and stage of change of the participant. Adapted motivational interviewing was used to help participants recognize and change unhealthy behavior. If applicable, quitting smoking was advised as the first treatment goal. Treatment for all risk factors that the participant was motivated for working on, had to start within three months. In case of several risk factors, these treatments could be combined within one visit. The first visit was advised to take at least 20 minutes, while the duration of follow-up visits and follow-up intervals could be adapted to patient preferences. Dependent on the specific individual risk factor(s), participants were offered self-monitoring for direct feedback. For example, all participants with overweight were offered a weighing scale and pedometer at home. The use of self-monitoring was free of charge.

For both groups, medication adjustments were made by the practice nurses under supervision of the GP. For each visit the practice nurses filled in a step by step treatment plan based on the Dutch GP guideline.

### Outcomes

After one year anthropometric data, information on smoking behavior, changes in medication and medical history, fasting blood glucose, lipids and creatinine were collected. The baseline questionnaire was repeated and treatment plans were analyzed on the number and duration of visits and use of self-monitoring. For the calculation of the SCORE risk assessment both at baseline and after one year, the age at baseline was used, entailing slight underestimation of the risk after one year.

### Statistical analysis

Based on two earlier studies, a difference between both study groups of 2% in SCORE risk assessment was assumed [24,25]. After screening of the first 82 participants in January 2009, the observed standard deviation was 3.3. Power analysis with a standard deviation of 4 revealed that 86 subjects in each study arm were needed for 90% power and 5% significance level 2-sided. To allow for drop out we aimed to enroll at least 200 participants.

Data were analyzed for all participants that attended the data collection after one year. Baseline characteristics in both treatment groups, characteristics of participants and drop-outs, and characteristics of the treatment in both groups were evaluated with unpaired Student’s t-test for continuous variables and with Mann–Whitney U test when non-parametric testing was indicated. Normality of data was assessed by visual analysis of histograms and QQ-plots. Categorical variables were analyzed with Fisher’s exact test 2-sided. Change in risk profile after one year was analyzed with paired t-test for continuous variables and McNemar test for dichotomous variables. To compare the effects after one year in both groups, unpaired t-test was used for continuous variables and the difference in proportions for the other variables. We used statistical package SPSS version 16. A p-value < .05 was considered significant.

## Results

Figure 1 shows the flow of patients through the SPRING study. The drop-out level was 11%; 6% in the control group and 15% in the intervention group (p = .045). Figure 1 also shows reasons for drop-out.

Table 3 shows that during baseline measurements both groups were well balanced with no significant differences between groups. All p-values were > .1, except for HDL cholesterol and total cholesterol/HDL ratio, which tended to be higher in the intervention group (p = .068 and p = .071 respectively).

**Table 3:** **Baseline characteristics for both treatment groups,*n*(%) unless otherwise indicated**

	**Control groupN = 90**	**Intervention groupN = 89**
**General characteristics:**		
Age (years), M (SD)	65 (5.7)	65 (5.3)
Men	61 (68%)	63 (71%)
Level of education (level 1–4)*		
Level 1 (%)	9%	10%
Level 2 (%)	40%	45%
Level 3 (%)	30%	24%
Level 4 (%)	22%	21%
**Medication use:**		
Medication for hypertension	23 (26%)	26 (29%)
Medication for hypercholesterolemia	10 (11%)	5 (6%)
**Cardiovascular risk factors:**		
Current smokers	30 (33%)	27 (30%)
Physically inactive participants	28 (32%)	23 (27%)
BMI (kg/m^2^), M (SD)	29 (4.0)	28 (3.3)
Waist circumference (cm), M (SD)	102 (10.8)	101 (8.0)
Syst. blood pressure (mmHg), M (SD)	158 (16.3)	158 (17.1)
Diast. blood pressure (mmHg), M (SD)	91 (8.5)	92 (9.5)
**Blood test (after overnight fast):**		
Total cholesterol (mmol/l), M (SD)	5.6 (.94)	5.6 (.85)
HDL cholesterol (mmol/l), M (SD)	1.3 (.34)	1.3 (.29)
LDL cholesterol (mmol/l), M (SD)	3.6 (.81)	3.6 (.78)
Triglycerides (mmol/l), median (P_25_ –P_75_)	1.4 (1.02-1.76)	1.4 (1.06-2.15)
Total cholesterol/HDL ratio, M (SD)	4.4 (1.11)	4.7 (1.11)
Glucose (mmol/l), M (SD)	5.4 (.68)	5.4 (.50)
Creatinine (mmol/l), M (SD)	91 (16.8)	89 (17.6)
**SCORE risk calculation:**		
SCORE (%), median (P_25_ –P_75_)	7.19 (5.23-10.64)	7.58 (6.08-10.63)
**Treatment indication:**		
Treatment goals/person, median (P_25_ –P_75_)	3 (3–4)	3 (3–4)
Overweight	86 (96 %)	87 (98 %)
Smoking	30 (33 %)	27 (30 %)
Physical inactivity	29 (32 %)	24 (27 %)
Hypertension	70 (78 %)	67 (75 %)
Hypercholesterolemia	73 (81 %)	69 (76 %)

inactive: physical activity < 30 min./day on 5 d./w, based on the SQUASH questionnaire, which was completed by 89 controls and 85 intervention participants. For missing data, treatment indication was based on the 2 questions about physical activity from the invitation letter.

* Level of education: 1 = no education or only primary education 2 = lower secondary education, 3 = higher secondary education, 4 = college or university.

We compared drop-outs with participants completing follow-up on these similar variables. No significant differences were found between drop-outs and participants, except for waist circumference (107 cm (SD 12) for drop-outs and 102 cm (SD 10) for participants, p = .011).

The treatment period (from the first visit to the practice nurse until the outcome measurements) lasted 345 days (SD 76 days), and was not different for intervention and control group (p = .81). The time between baseline measurements/ randomization and first treatment visit was 97 days (median, P_25_ –P_75_: 65–125) and was not different between both groups (p = .25).

The mean number of visits was 2.6 (SD 1.5) for the control group and 4.9 (SD 2.2) for the intervention group (p < .001) and the median duration of each visit was 27 (P_25_ –P_75_:20–33) minutes/visit for the intervention group and 23 (P_25_ –P_75_:19–30) for the control group (p = .048).

In the intervention group 15.7% (14/89) received new/more medication for hypercholesterolemia, compared to 8.9% (8/90) of the control group. In the intervention group 32.6% (29/89) received new/more medication for hypertension, compared to 18.9% (17/90) of the control group. The difference in medication prescription was not significant for cholesterol (p = .18), but was significant for hypertension (p = .041).

Table 4 shows that for both groups the SCORE risk assessment dropped significantly after one year, however the difference between both treatment groups was not significant. The effect on the SCORE assessment was also evaluated using risk category reclassification. In both groups almost 50% of the participants remained in the same risk category, 44% improved and 8% deteriorated. From the analysis of the risk reclassification again no significant difference between both groups was found. Most separate risk factors tended to improve after one year in both groups, except glucose. Only for waist circumference, the intervention group showed a significantly larger improvement than the control group.

**Table 4:** Effect on end points after one year of treatment

	**Control groupN = 90**	**Intervention groupN = 89**	**Difference betweengroups (95% CI)**
**SCORE risk assessment:**			
SCORE (%)	−1.6 (−2.2– -1.0)*	−1.8 (−2.4– -1.2)*	.2 (−.6–1.1)
**Risk reclassification:**			
No change	42 (46.7 %)	44 (49.4 %)	2.7 (−11.9–17.3)
Improvement	41 (45.6 %)	37 (41.6 %)	4.0 (−10.5–18.5)
Deterioration	7 (7.8 %)	8 (9.0 %)	1.2 (−6.9–9.3)
**Cardiovascular risk factors:**			
Current smokers (Δ%)	−8.9 (−16.8– -3.9)*†	−11.2 (−19.7– -5.5)*†	2.3 (−6.5–11.1)
Phys. inactive participants (Δ%)	−4.5 (−11.2– -1.3)†	−11.8 (−20.6– -5.8)†	7.3 (−.8–15.4)
BMI (kg/m^2^)	-.1 (−.41–.28)	-.1 (−.38– .17)	.04 (−.4–.5)
Waist circumference (cm)	−1.9 (−3.1– -.67)*	−3.7 (−4.8– -2.6)*	1.8 (.2–3.4)*
Syst. blood pressure (mmHg)	−5.6 (−8.5– -2.6)*	−6.8 (−10.3– -3.2)*	1.2 (−3.4–5.8)
Diast. blood pressure (mmHg)	−3.3 (−4.8– -1.8)*	−4.4 (−6.3– -2.4)*	1.1 (−1.4–3.5)
**Blood test:**			
Total cholesterol (mmol/l)	-.14 (−.31–.03)	-.32 (−.52– -.11)*	.17 (−.09–.44)
HDL cholesterol (mmol/l)	.10 (.05–.14)*	.07 (.03–.11)*	.02 (−.04–.08)
LDL cholesterol (mmol/l)	-.18 (−.34– -.02)*	-.34 (−.52– -.16)*	.16 (−.08–.39)
Triglycerides (mmol/l)	-.07 (−.18–.04)	-.17 (−.32– -.03)*	.10 (−.08–.28)
Tot. cholesterol/HDL ratio	-.34 (−.50– -.18)*	-.52 (−.73– -.32)*	.18 (−.08–.44)
Glucose (mmol/l)	.28 (.15–.41)*	.17 (.06–.29)*	.11 (−.07–.28)
Creatinine (mmol/l)	−6.0(−8.2– -3.7)*	−5.1 (−7.2– -2.9)*	-.9 (−4.0–2.2)

* significant

† McNemar’s test in dichotomous variables: Δ% current smokers: control group p = .008, intervention group p = .006; Δ% physically inactive participants: control group p = .54, intervention group p = .052.The questionnaire about physical activity after one year was completed by 89 participants from the control group and 89 participants from the intervention group. The 85 participants from the intervention group and the 88 participants from the control group that completed the questionnaire twice were analyzed.

Effects are indicated as delta values in continuous variables and Δ % in dichotomous variables, both with 95% CI. Risk reclassification is indicated as *n* (%) in both groups and as % in difference between groups.

## Discussion

### Main findings

The goal of the SPRING study was to investigate the effect of one year of risk management performed by practice nurses, using intensive counseling and home monitoring devices, on cardiovascular risk. The results of the study show that there was no significant additional effect of this intervention compared to standard treatment by practice nurses, despite investment of extra time. For both groups the SCORE risk assessment dropped similarly and significantly. The effect size is comparable to a decrease in systolic blood pressure from 160 to 120 mmHg for a non-smoking 60-year old woman with unchanged lipid levels.

### Comparison with existing literature

The positive effect that was achieved in both study groups after treatment of risk factors by practice nurses corresponds with previous research on cardiovascular risk management by practice nurses [5-7]. Effective treatment and follow-up are increasingly necessary as improved screening strategies like the recently introduced Dutch Prevention Consultation and similar initiatives elsewhere, such as the NHS Health Checks, have been developed [26,27]. Because of these screening initiatives, increasing numbers of individuals with an identified elevated cardiovascular risk are expected.

Although most risk factors tended to improve after one year of treatment, this was not the case for fasting glucose levels and body mass index (BMI). A possible explanation is that the participants belong to a middle aged, adipose population with an elevated risk of glucose intolerance. After one year the glucose might rise slightly due to increase of age. Besides, the focus of this study was on combined cardiovascular risk and not on weight only [28]. Another explanation is the prescription of antihypertensive medication; beta blocking agents and diuretics increase the risk of incident diabetes [29].

In the intervention group antihypertensives were more often newly added or the dosage was increased, compared to the control group. A similar trend was present for cholesterol lowering medication. The treatment programs for hypertension and hypercholesterolemia were the same for both groups, except for the use of home monitoring in blood pressure management for the intervention group. However, *decreased* use of medication in the intervention group might have been expected, because of reduced white coat effect and more feedback and subsequent adherence to therapy [12]. One possible explanation is that the target blood pressure values were 135/85 mmHg for home measured values in this study, compared to 140/90 mmHg for office measured values. Another explanation is that during visits for lifestyle counseling in the intervention group, blood pressure and lipid levels could also be discussed. As a consequence, medication may have been adjusted more frequently.

### Strengths and limitations

As far as we know, this is the first study that investigated the effect of combined pro-active counseling aided by self-monitoring devices by practice nurses in general practice.

Some circumstances may explain the decrease in SCORE assessment in both groups and the minor differences that were found between both study groups. First, we randomized at patient level instead of practice level, with the result that participants from both groups were treated by the same practice nurses. Randomization at patient level was chosen to diminish the influence of differences between practices c.q. practice nurses. Another advantage was that it made the study more efficient, since individuals were the unit of analysis and only a limited number of practices was required with this design. All practice nurses had followed the training and were very motivated. The fact that the time investment in the intervention group was much higher, made us expect that contamination of research conditions would be limited. However, some degree of contamination might be present and may have diminished the difference between both groups.

Second, participants in both treatment groups were aware of the study goals and were probably more motivated than average patients.

For example the remarkably large decrease in proportions of smokers in both groups suggests the influence of these factors.

The intervention was an integrated combination of self-monitoring and a more pro-active and motivational interviewing-based approach. The two group design made it impossible to determine the separate influence of these specific aspects of the intervention.

The drop-out rate was 6% in the control group and 15% in the intervention group. The reasons for drop-out as represented in Figure 1 do not indicate that the intensity of the program has been the main reason for increased drop-out in the intervention group. However, the precise extent to which the intensity of the program contributed to the dropping-out of persons with non-cardiovascular comorbidity, family related circumstances or lack of motivation in the intervention group is unknown, although the intensity of the intervention program was intended to be adjusted in accordance with the participants’ preferences, to optimize involvement and motivation.

During the screening procedure a considerable number of individuals was excluded, due to the extensive amount of information that needed to be collected for identifying individuals at a moderately elevated cardiovascular risk. We nevertheless expect our results to be applicable for a primary care population with an indication for cardiovascular risk management.

During the screening we used a risk calculator, provided by the Dutch GP Society. For the analyses we used syntax to calculate SCORE with 2 decimals accuracy. Using this latter calculation, 10 (11%) participants from the intervention group and 16 (18%) from the control group appeared to have a baseline risk <5%. Normally treatment is based on paper-based risk charts or the calculator we used, so our selection represents the patients that would also be identified during normal practice.

## Conclusions

Based on this study, we confirm previous findings that practice nurses in general practice are well equipped for their task to treat and counsel individuals with an elevated cardiovascular risk. However, the intervention group did not achieve a better effect on cardiovascular risk, which suggests that the treatment program based on self-monitoring should not be directly implemented into daily practice. The time investment is greater in such a program and the number of prescribed medications tends to be higher, without any relevant additional effect, but with subsequent higher risk of side effects. Factors determining the effect of self-monitoring have to be further unveiled, as well as consequences for health care costs and long term effects on morbidity and mortality.

## Abbreviations

GP, General Practitioner; HDL/LDL, High/Low Density Lipoprotein; BMI, Body Mass Index: weight (kg)/ square-length (m^2^); SCORE, Systematic Coronary Risk Evaluation: 10-year risk of fatal cardiovascular disease; TSH, Thyroid-stimulating Hormone; T4, Thyroxin.

## Competing interest

The authors declare that they have no competing interests.

## Author’s contributions

AS, JB, KM and AT designed the study, AT collected the data, AT and KG analyzed and interpreted the data, AT drafted the article, all other authors revised it and all authors gave final approval of the version to be published.

## Pre-publication history

The pre-publication history for this paper can be accessed here:

http://www.biomedcentral.com/1471-2296/13/90/prepub
